# *Plasmodium falciparum*: Differential Selection of Drug Resistance Alleles in Contiguous Urban and Peri-Urban Areas of Brazzaville, Republic of Congo

**DOI:** 10.1371/journal.pone.0023430

**Published:** 2011-08-15

**Authors:** Yoko Tsumori, Mathieu Ndounga, Toshihiko Sunahara, Nozomi Hayashida, Megumi Inoue, Shusuke Nakazawa, Prisca Casimiro, Rie Isozumi, Haruki Uemura, Kazuyuki Tanabe, Osamu Kaneko, Richard Culleton

**Affiliations:** 1 Department of Protozoology, Institute of Tropical Medicine (NEKKEN), and the Global COE Program, Nagasaki University, Nagasaki, Japan; 2 Centre d’Etudes des Resources Vegetales, Brazzaville, Republic of Congo; 3 Department of International Health, Institute of Tropical Medicine (NEKKEN), Nagasaki University, Nagasaki, Japan; 4 Laboratory of Malariology, International Research Centre of Infectious Diseases, Research Institute of Microbial Diseases, Osaka University, Osaka, Japan; 5 Malaria Unit, Institute of Tropical Medicine (NEKKEN), Nagasaki University, Nagasaki, Japan; Université Pierre et Marie Curie, France

## Abstract

The African continent is currently experiencing rapid population growth, with rising urbanization increasing the percentage of the population living in large towns and cities. We studied the impact of the degree of urbanization on the population genetics of *Plasmodium falciparum* in urban and peri-urban areas in and around the city of Brazzaville, Republic of Congo. This field setting, which incorporates local health centers situated in areas of varying urbanization, is of interest as it allows the characterization of malaria parasites from areas where the human, parasite, and mosquito populations are shared, but where differences in the degree of urbanization (leading to dramatic differences in transmission intensity) cause the pattern of malaria transmission to differ greatly. We have investigated how these differences in transmission intensity affect parasite genetic diversity, including the amount of genetic polymorphism in each area, the degree of linkage disequilibrium within the populations, and the prevalence and frequency of drug resistance markers. To determine parasite population structure, heterozygosity and linkage disequilibrium, we typed eight microsatellite markers and performed haplotype analysis of the *msp1* gene by PCR. Mutations known to be associated with resistance to the antimalarial drugs chloroquine and pyrimethamine were determined by sequencing the relevant portions of the *crt* and *dhfr* genes, respectively. We found that parasite genetic diversity was comparable between the two sites, with high levels of polymorphism being maintained in both areas despite dramatic differences in transmission intensity. Crucially, we found that the frequencies of genetic markers of drug resistance against pyrimethamine and chloroquine differed significantly between the sites, indicative of differing selection pressures in the two areas.

## Introduction

Africa’s population is expected to triple by 2050, when it is estimated that 800 million people (half the population) will reside in urban areas [1], with the most significant urbanization occurring in West Africa, where 66% of the population will live in urban areas in 10–15 years time [2]. In 2004, the Pretoria Statement on Urban Malaria was issued. It declares that “Urban malaria in sub-Saharan Africa is a major health problem and is likely to increase in importance unless addressed” [2]. Currently, relatively little is known about the consequences of urbanization for malaria epidemiology, even though it is likely to become significantly more important in the coming years. Certainly, it is likely that transmission rates and parasite prevalences in urban areas are lower than in peri-urban and rural areas, due, for the most part, to lower entomological inoculation rates associated with reduced vectorial capacities [3], [4]. However, the consequences of this reduced transmission for parasite genetic diversity, the acquisition of host immunity and the selection and spread of parasite drug resistance remain unclear. Previous studies comparing these factors between areas of varying transmission intensities have usually done so using data from non-contiguous areas. In reality, the process of urbanization in sub-Saharan Africa creates islands of low transmission areas surrounded by regions of higher transmission, so that in order to understand how the expansion of urban centres into the surrounding peri-urban regions will impact on malaria transmission and epidemiology, it is important to compare parasite populations in contiguous peri-urban and urban areas.

The relationship between transmission rates and parasite genetic diversity is important, as the rate of acquisition of immunity against a parasite population depends to some degree on both these factors. It has been shown, for example, that immunity to malaria is strain-specific [5], [6], [7], [8], and that increased transmission leads to an increase in the number of strains in circulation within a population [9], [10], [11]. There are a limited number of reports that describe the relationships between transmission rates and parasite genetic diversity, and these have produced conflicting conclusions. Analyses conducted in Senegal [12], Tanzania and Sudan [13] and Papau New Guinea and Tanzania [14] demonstrate significantly lower multiplicities of infection (MOI) and parasite population genetic diversity in low transmission areas compared to high transmission areas. Conversely, studies conducted in Tanzania [15], West Uganda [16], Burkina Faso [17] and Papua New Guinea [18] report no correlation between transmission intensity and MOI and parasite genetic diversity. In all these cases, the high and low transmission areas were non-contiguously located, often separated by hundreds of kilometres, and in two cases were situated in different countries. In this study, we investigate the degree of parasite genetic diversity and MOI from contiguous areas of high and low transmission.

How urbanization will affect the selection and spread of drug resistance is currently very poorly understood. However, the relationships between transmission intensity and drug resistance has been the subject of theoretical modelling and some field studies, again, with conflicting results. Theoretical models predominantly propose three main potential ways that transmission intensity can influence the spread of drug resistance; i) resistance is predominantly selected when transmission intensity is high [19], ii) resistance is predominantly selected when transmission intensity is low [20], [21], [22], or resistance is predominantly selected at high and low transmission intensities, with less selective pressure at intermediate levels [23], [24], [25]. The relationships between transmission intensity and drug resistance selection pressure are complex, and involve many factors including within-host competition between parasites [24], fitness costs of drug resistance [26], the consequences of host immunity [27], levels of community drug use [28], and parasite recombination rates [24], [29], [30]. Empirical data that support these models is scarce. The way in which chloroquine (CQ) and sulphadoxine/pyrimethamine (SP) resistance first arose in areas of low transmission intensity before spreading world-wide [31], [32], [33], suggests that low transmission intensity conditions may be optimal for the selection of drug resistance, at least *de novo*. In Uganda, Talisuna *et al* (2007) describe a situation in which the highest levels of resistance to both CQ and SP, as measured by treatment failure and the prevalences of molecular markers of resistance, were always higher in the highest transmission areas. For CQ, resistance was also high at the lowest transmission rates, and was at its lowest in areas of intermediate transmission intensity. This was not the case for SP resistance, for which levels of resistance were proportional to transmission intensities [34].

Here, we investigate the relationships between transmission intensity, parasite genetic diversity and the selection of drug resistance in contiguous urban and peri-urban regions of Brazzaville, Republic of Congo, in an attempt to better understand the role of urbanization on malaria epidemiology.

## Materials and Methods

### Sample collection

A total of 356 blood samples were collected from all patients visiting two health centers within the city of Brazzaville, Republic of Congo between mid August and mid November 2005 corresponding to the beginning of the wet season. There were no age restrictions, and all patients were sampled, regardless of their symptoms. Blood (∼50 µl) was applied to Whatman® FTA® Classic Filter Paper cards (Whatman®, USA) *via* finger prick, and left to air-dry. Thick blood smears were also prepared for each sample, stained with Giemsa’s solution, and examined on-site by an experienced microscopist. All patients provided data on their age, sex, clinical symptoms before presentation at the health center, region of habitation (whether urban or peri-urban), and which type (if any) anti-malarial drug treatment had been used prior to presentation. Patients were number coded to preserve anonymity. A further 150 samples were collected from a health centre near Pointe-Noire, a city on the west coast of the country, approximately 380 km from Brazzaville, and 141 from a health centre near Gamboma, a town approximately 250 km to the North of Brazzaville, in 2006. These samples were collected on Whatman® 31ETCHR filter paper. RI. Blood spots on filter paper were shipped to Japan in November 2005 (Brazzaville) or November 2006 (Pointe-Noire and Gamboma), stored in individual plastic bags kept at 4°C and DNA was extracted within 2 months of their arrival. Ethics approval for these collections was obtained from the Osaka University Committee for Ethics in Scientific Research, Osaka University, Osaka, Japan, and sampling was authorized by the administrative authority of the Ministry for Research and Ministry for Health in the Republic of Congo. Written informed consent was obtained from individual patients (or their legal guardians), and anti-malarial treatment was provided when appropriate

### Study site, population, and classification of urban and peri-urban districts

**Figure 1:**
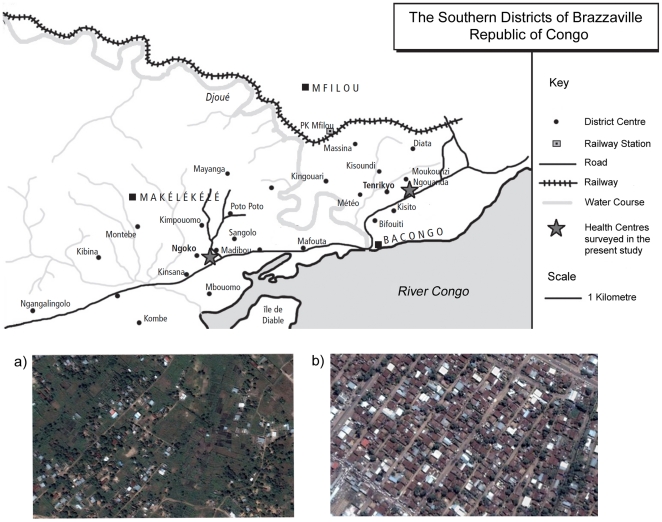
Map of the study area in Brazzaville, Republic of Congo. Blood sample donors were recruited from two health centres, Madibou, which serves patients from the peri-urban area to the west of the Djoue river, and Tenrikyo, which predominantly serves those resident in the urban area to the east of the Djoue. Both centres are marked with a star symbol. Insets (a) and (b) show aerial photographs typical of the regions in which patients from the peri-urban (a) and urban (b) areas reside.

Brazzaville, the capital of the Republic of Congo, has a population of about 1,000,000. It has a humid tropical climate with two seasons; the rainy season, which begins in September and lasts until May, is characterized by abundant rainfall and violent evening storms. During the dry season, which begins in June and ends in September, there is practically no rain at all [35]. The main malaria vector is *Anopheles gambiae*. The two health centers from which samples were collected for this study (Tenrikyo and Madibou) are located 8 km apart within the southern district of Brazzaville (Figure 1). Tenrikyo health center is situated within the urban region of the district of Makélékélé, and provides health services for patients from the urban regions (*i.e.* areas with a population of more than 1000 persons per km^2^, as defined by Hay *et al* (2005)[36] of Makélékélé, Bacongo and Mfilo. Occasionally, patients form the peri-urban area of Makélékélé (Poto-Poto Djoué) also present here. Madibou health center is situated in the peri-urban region of Makélékélé, and serves patients exclusively from peri-urban areas (*i.e.* areas with populations of between 250 and 1000 persons per km^2^, as defined by Hay *et al*, 2005) Patients from both health centers were asked to identify the districts in which they lived, and were assigned to either the “urban” or the “peri-urban” group. Two hundred and two blood samples were collected from Madibou health center, and 154 from Tenrikyo.

The urban and peri-urban districts from which patients were recruited are in very close proximity to each other, in places separated by only a few hundred meters, and the parasitological, epidemiological and entomological differences between them were previously described in detail by Trape *et al* (1987) [35], [37], [38], [39], [40]. The urban areas are characterized by low malaria transmission rates (2–12 infective mosquito bites per person per year [37]) and meso-endemicity (parasite prevalences of ∼39%, as determined by active case detection of school children within Bacongo in 1984 [37], and the peri-urban areas by high transmission rates (∼50 infective mosquito bites per person per year [40]) and hyper-endemicity (parasite prevalences of >80%, as determined by active case detection of school children resident in the peri-urban area of Makélékélé [39]). Although these entomological inoculation rates and parasite prevalences were determined 20 years prior to the present study, they fit well with the data presented in the present work (see parasite prevalence in the results section), and with those published in 2008 [41].

### Parasite DNA extraction from blood spots

For the malaria parasite 18s RNA gene species identification PCR performed in order to detect and species-type malaria parasites, PCR was performed directly on discs cut from the blood sample (according to the manufacturer’s instructions), for those samples from Madibou and Tenrikyo health centers that were collected on Whatman® FTA® cards. Briefly, a disc of 1.2 mm in diameter was punched from the centre of each dried blood spotted card and washed three times with Whatman® FTA® Purification Reagent, and twice with TE buffer. This treated disc was then used directly in subsequent PCR analyses. Subsequently, for all further PCR and sequencing analyses, DNA was extracted from the remaining blood spot using an EZ1 BioRobot™ (QIAGEN, Hilden, Germany) according to the manufacturer's instructions. For samples from Pointe-Noire and Gamboma collected on Whatman® 31ETCHR filter paper, DNA extraction was performed using the EZ1 BioRobot™ (QIAGEN, Hilden, Germany) as above.

### Species typing PCR

In order to detect the presence of malaria parasites in the samples, and to determine which species of parasites were present, a nested PCR was performed targeting the malaria parasite 18s RNA gene, as previously described [42]. This analysis was performed for all 647 samples. We estimate that the maximum sensitivity of our PCR assay was one parasite per µl of blood.

### Correlation between microscopy and PCR

The correlation between the PCR diagnosis and that achieved by thick smear microscopy on site was performed using Kappa co-efficient analysis for all samples from Brazzaville. Sub-microscopic parasite carriers were identified as those individuals deemed negative by miscroscopy, but with positive PCR results.

### Typing of MSP1 blocks 2, 4a, 4b, and 6

*P. falciparum msp1* may be divided into 17 distinct blocks. We typed four of these blocks; blocks 4a 4b and 6, which are dimorphic, and block 2, which is trimorphic. There are, therefore, 24 possible haplotype combinations for these four blocks. We typed 61 isolates from the peri-urban area, and 41 isolates from the urban area at these four blocks using a PCR protocol which involves the use of primers specific for each allelic type, as previously described [43]. Briefly, forward and reverse primers corresponding to allelic type specific- and inter-allele conserved sequences in blocks 2, 3, 4a, 4b, 5 and 6 were used to differentiate all possible 24 allelic haplotypes covering variable blocks 2, 4a, 4b and 6. Following amplification of blocks 2 to 6, nested PCR was performed to determine allelic types in blocks 4a and 4b. TaKaRa LA Taq™ polymerase (Takara, Japan) was used for amplification of block 2–6, and AmpliTaq Gold® polymerase (Applied Biosystems, USA) for typing of other blocks. PCR products were visualized under UV transillumination following electrophoresis on 2agarose gels stained with ethidium bromide. This allowed us to estimate the minimum number of clones per infection (multiplicity of infection (MOI)), as well as providing data on the diversity of MSP1 within these populations. We also performed this analysis for 23 samples from Pointe-Noire and 27 samples from Gamboma for comparison to the Brazzaville population.

### Sequencing of *crt* and *dhfr* genes

Mutations associated with parasite drug resistance to pyrimethamine and chloroquine were assessed by direct sequencing of the *dhfr* and *crt* genes respectively. *dhfr* alleles were determined for 46 and 54 of the samples from the urban and peri-urban areas respectively. *crt* alleles were determined for 37 (urban) and 46 (peri-urban) of the samples. We also sequenced the *dhfr* gene for 24 of the samples from Pointe-Noire and 23 of the samples from Gamboma for comparison to the Brazzaville population. PCR amplification of gene fragments containing point mutations associated with drug resistance was performed for each gene as previously described [44]. For *dhfr*, mutations were assessed at amino acid positions 51, 59, 108 and 164. For *crt*, mutations at amino acid positions 72, 73, 74, 75 and 76 were assessed. Mixed infections were identified in those samples that had double peaks at the relevant nucleotide mutation sites as assessed by visualization of electropherogram peaks following sequencing. In order to meet our criterion for a mixed infection, minor peaks were ignored if they were less than 10% the peak height of the major peak.

### Estimation of allele frequencies of *crt* and *dhfr*

In order to estimate allele frequencies for *crt* and *dhfr* wild type and mutant alleles, we utilized the computer program MalHaploFreq [45]. This program allows the estimation of allele frequencies from prevalence data. As high numbers of mixed infections are associated with areas of high transmission, the prevalences of mutant and wild type alleles in a population of samples may not accurately reflect the true frequencies of these alleles in the parasite population. The program uses a maximum likelihood algorithm to estimate allele frequencies based on prevalence data combined with the number of clones per sample (MOI, which we determined by haplotyping *msp1*). In order to assess the statistical significance of allele frequencies between the urban and peri-urban areas, likelihood ratio tests were performed for each area separately, and for the combined data set. The likelihood ratio statistic was then computed as being twice the difference of the sum of the log-likelihoods from the independent likelihood ratio tests and the log-likelihood of the combined data set.

### Analysis of eight putatively neutral microsatellite markers

To assess the population structure of the parasite population(s) considered here, and to determine whether parasites from the urban and peri-urban areas were in panmixia, eight putatively neutral microsatellite markers were typed for 41 samples from the urban area, and 39 from the peri-urban area. The microsatellite marker names, accession numbers, whether they are located within coding regions or non-coding regions, and chromosomal locations are summarized in Table 1. Analysis was performed according to Greenhouse *et al.* (2006)[46] or Anderson *et al* (1999)[47], depending on the marker. Briefly, one primer was labeled at the 5' end with either a HEX or 6-FAM fluorophore for detection in Applied Biosystems 3730 DNA Analyzer with GeneMapper Software (Applied Biosystems). The loci of TA81, Poly alpha, TA53, TA43 and TA17 were analyzed by semi-nested PCR using primers of Anderson *et al* (1999). For TA40, TA60 and PfPK2, primers were as designed by Greenhouse *et al* (2006) for single-round PCR. In case of PfPK2, the Greenhouse *et al* primer pair was used in the second round of a nested PCR following initial amplification with the primers of Anderson *et al* (2006).

**Table 1:** Microsatellite markers used in this study. *h*  =  haplotypes diversity index, *P*-values determined by t-tests.

Microsatellite marker	Chromosome	Accession number	*h* (urban)	*h* (peri-urban)	*P*-value	*Ps (p-value)* *Urban Vs Peri-urban*
Poly-α	4	LI8785	0.894	0.913	0.55	0.70 (0.60)
TA81	5	AF010510	0.853	0.856	0.98	0.64 (0.19)
TA40	10	AF010542	0.925	0.942	0.75	0.55 (0.37)
PK2	12	X63648	0.879	0.903	0.85	0.72 (0.43)
TA60	13	AF010556	0.837	0.878	0.63	0.71 (0.46)
TA43	14	AF010544	0.931	0.923	0.73	0.64 (0.40)
TA53	9	AF010552	0.664	0.583	0.32	0.80 (0.24)
TA17	8	AF010531	0.822	0.844	0.64	0.70 (0.24)

*Ps*  =  percentage similarity, percent rank (from the smallest) of the observed value among 2000 permutation results was used as a one-tailed P-value.

### Population genetic analyses and statistical treatments

Parametric statistical tests were used when data were shown to be normally distributed according to Anderson-Darling tests for normality. In the case of non-normally distributed data, non-parametric tests were used. The statistical significance of differences in parasite prevalence and the proportion of sub-microscopic carriers between areas were tested using Chi-squared tests with Yates’ correction for data sets less than 10. The statistical significance of differences in MOI between areas, as determined by *msp1* haplotyping, was analyzed using Mann-Whitney U-tests. The mean ages of patients carrying sub-microscopic parasite infections, and the mean parasite density of patients from the two areas were compared using Student’s two-tailed t-tests. These tests were performed using MINITAB® software v15 (LEAD Technologies, Inc., UK). The haplotype diversity index (*h*) for *msp1* for parasites from the urban and peri-urban areas was calculated using the formula *h*  =  {n/(n – 1)} 5{1–∑ pi^2^} (Nei, 1987) where p and i are the frequency and number of *msp1* haplotypes, respectively. The variance (*V*) of *h* was calculated using the formula *V*  =  {2/n(n – 1)}[2(n – 2){ ∑ pi^3^ - (∑pi^2^)^2^} + ∑pi^2^ - (∑pi^2^)^2^] [43]. Statistical differences between values of *h* for *msp1* were determined by Student’s two-tailed t-tests. This method was also applied to determine *h* for each of the eight microsatellite markers. To quantify the pairwise similarity of haplotype pattern between two sites, percent similarity (Ps) was calculated as:

Ps  =  {SIGMA_for_i} [ Minimum (PiA, PiB)]where PiA and PiB are frequency of ith haplotype in Site A and Site B, respectively. The statistical significance of Ps was evaluated by comparing observed Ps to that calculated for randomly mixed samples by 2000 permutations. Percent rank (from the smallest) of the observed value among the 2000 permutation results was used as one-tailed P-value. Linkage disequilibrium within the urban and peri-urban populations was measured using LIAN v3.5 [48]. This program computes the standardized index of association (*I^S^_A_*), a measure of haplotype-wide linkage [49]; *P*-values were determined by a Monte Carlo simulation process, with 100,000 iterations. Only those samples for which a complete set of microsatellite alleles were scored were used for this analysis. Genetic differentiation between populations based on the microsatellite data set was measured by calculating values of *F_ST_,* carried out using FSTAT v.2.9.4 [50], [51]. *P-*values for *F_ST_* were calculated by running 10,000 randomizations in a population differentiation test assuming random mating within populations. FSTAT v.2.9.4 was also used to search for linkage disequilibrium between pairs of microsatellite loci for each area. The majority of our samples contained multiple alleles at numerous loci. For the *F_ST_* and linkage disequilibrium analyses, the major allele at each locus (in the case of multi-clonal infections) was designated the “major” allele, and haplotypes were constructed using these alleles only, as this approach has previously been shown to give very similar results to comparisons of data sets in which multiple infections are removed [52]. The effective populations sizes of the parasite population from the urban and peri-urban areas was determined using an infinite alleles model where population size (*N_e_*) is determined by the relationship *N_e_*µ  =  *h*/4(1 - *h*), where µ, the mutation rate, was taken as 1.59 5 10^−4^ (95% confidence interval: 6.98 510^−5^, 3.7 5 10^−4^), as calculated by Anderson *et al* (2000) from work previously conducted in which the progeny of a laboratory cross of *P. falciparum* were typed with large numbers of microsatellite markers, and the frequency of non-inherited markers scored [53].

## Results

### Clinical differences between malaria patients from urban and peri-urban areas

There were no significant differences in the ages or sex of patients presenting to either health center, regardless of their area of residence. There were clear differences in parasite prevalences between those patients residing in the urban areas, and those residing the peri-urban area (Table 2). By microscopy, 37% of patients presenting to either health center or who were resident in the urban area were positive for *P. falciparum*, compared to 59% of those resident in the peri-urban regions. When PCR diagnosis was performed, these percentages rose to 42% *P. falciparum* positive for the urban residents, and 75% positive for the peri-urban residents. The correlation between microscopy and PCR were assessed using Kappa co-efficient tests, which revealed a substantial correlation for peri-urban residents (K = 0.71; 95% confidence interval (CI), 0.61–0.81) and an almost perfect correlation for urban residents (K = 0.87; 95% CI, 0.79–0.95), indicating a larger percentage of sub-microscopic parasite carriers in the peri-urban region (20% of all *P. falciparum* cases were sub-microscopic in the peri-urban area, compared with 11% in the urban area, *p*<0.001, (chi-squared test, with Yate’s correction). Combining both areas, the mean age of sub-microscopic parasite carriers was significantly higher (15.9 years old) than that of patients with microscopically detectable parasitaemia (8.6 years old) (Student’s two-tailed t-test, *P* = 0.003, d.f. = 167), indicative of an age-associated acquisition of immunity. The mean age of sub-microscopic parasite carriers in the urban area was 21.0 years old compared to 15.4 years old in the peri-urban area, but this was not significantly different (Student’s two-tailed t-test, *P* = 0.42, d.f. = 12). The mean parasite density of those patients positive by microscopy presenting to the two health centers was 80,206 parasites per microlitre of blood for peri-urban residents, and 31,766 parasites per microlitre for urban residents, a statistically significant difference (Student’s two-tailed t-test, *P* = 0.048 d.f. = 104). There was no significant difference between the body temperatures of microscopically malaria positive patients from either area (Table 2).

**Table 2:** Clinical and Entomological data for both the urban and peri-urban regions.

Area	Urban	Peri-urban	Statistical significance
Entomological Inoculation Rate (EIR)^1^	2–12 ib/p/a	50 ib/p/a	ND
Percentage of parasite positive patients infected with;			
*P.falciparum*	100%	100%	ND
*P.vivax*	0.0%	0.0%	ND
*P.malariae*	0.0%	2.9%	ND
*P.ovale*	0.0%	0.7%	ND
Parasite prevalence (microscopy)^2^	37%	59%	*p*<0.001
Parasite prevalence (PCR)^2^	42%	75%	*p*<0.001
Sub-microscopic positive patients^2^	11%	20%	*p*<0.001
Mean age of sub-microscopic parasite carriers^3^	20.0 yr.	15.4 yr.	*p* = 0.048
Mean parasite density at the time of admittance^3, 4^	31,766/µL	80,206/µL	*p* = 0.048
Mean body temperature at the time of admittance^3, 4^	37.4°C	37.6°C	NS

1 data from Trape & Zoulani (1987).

2 *p*-values determined with chi-squared tests with Yate’s correction.

3 *p*-values determined with student’s two-tailed t-tests.

4 considers only those patients with parasitaemias detectable by microscopy.

ND  =  Not Determined, NS  =  Not Significant.

### Drug usage amongst febrile patients from the urban and peri-urban regions

At the time of admittance to either of the health centers, patients with febrile symptoms were asked to provide details of any anti-malarial drugs they had taken following the appearance of symptoms. 25.4% of febrile patients residing in the urban area had taken some form of anti-malarial treatment compared to 19.7% of the residents of the peri-urban region. There were no differences in the types of anti-malarial drugs taken by patients from either area, with the exception of quinine use, which was more commonly used in the peri-urban area than in the urban area (Table 3).

**Table 3:** Drug usage amongst all febrile patients from the urban and peri-urban regions presenting at health centers.

Area	Urban	Peri-urban
Percentage of patients taking malaria drugs	19.7%	25.4%
Percentage of Anti-malarial drugs taken by patients		
Chloroquine	58.8%	54.2%
Sulfadoxine-Pyrimethamine	8.85%	6.3%
Artemether	2.9%	4.2%
Amodiaquine	11.8%	10.4%
Quinine	8.9%	27.0%
Artemsinin and piperaquine combination	2.9%	0.0%
Unknown	8.9%	0.0%

### Multiplicity of infection as assessed by msp1 haplotyping and microsatellite analysis

**Figure 2:**
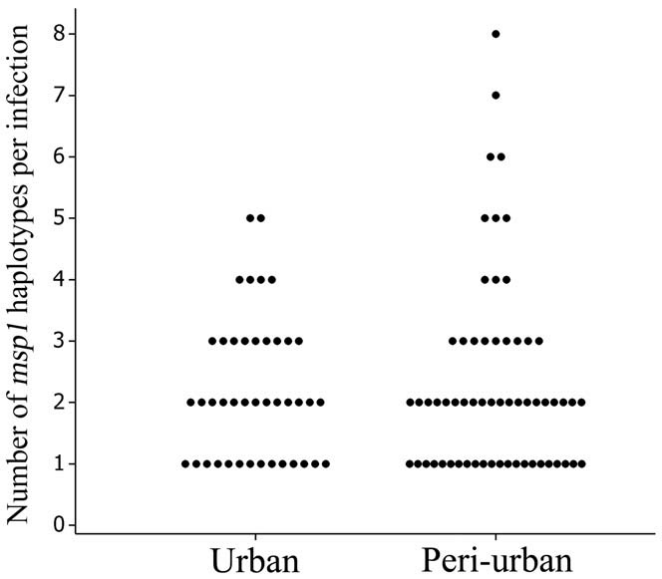
Multiplicities of infections (MOI) for the urban (n = 42) and peri-urban (n = 61) areas as assessed by *msp1* haplotyping.

The number of clones per infection was determined for 61 patients resident in the peri-urban area, and 42 patients from the urban area by assessing the numbers of individual *msp1* haplotypes by PCR (Figure 2). The mean number of *msp1* haplotypes per infection for urban residents was 2.21 (95% CI  = 1.86–2.56) compared to 2.34 (95% CI  = 1.94–2.74) for those resident in peri-urban area (Mann-Whitney test, *P* = 0.84). However, the maximum number of distinct haplotypes per patient was eight for peri-urban residents and five for urban residents. Multiplicities of infections (MOI) were also determined using microsatellite markers, where multiple infections were scored when more than one peak was observed for any of the eight markers assayed for each infection. The MOI for urban residents was 2.11 by this method (compared to 2.17 by *msp1* haplotyping for the same subset of samples), and 1.86 (2.04 by *msp1*) for the peri-urban residents. The strength of agreement between the two methods were assessed using weighted Kappa co-efficient analyses [54], which revealed a “fair” agreement (K = 0.22) between the methods.

### Evaluation of parasite genetic diversity between the urban and suburban areas based on msp1 typing

**Figure 3:**
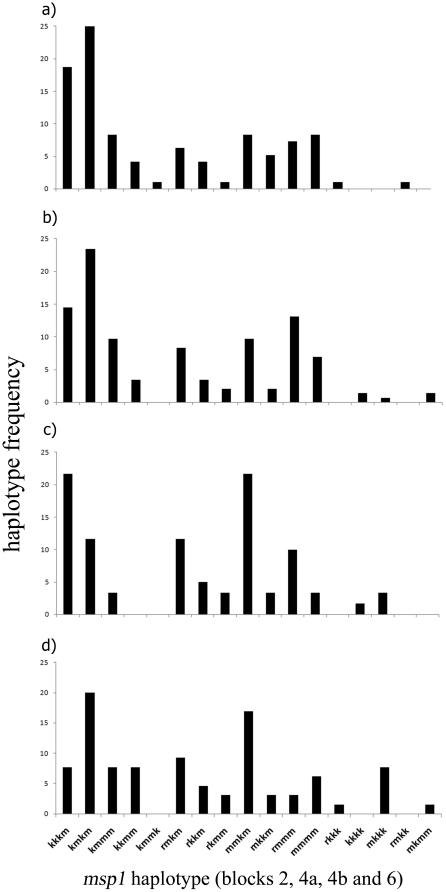
*msp1* haplotype frequencies of *Plasmodium falciparum* parasites in the urban (a) and peri-urban (b) areas of Brazzaville, and in Gamboma (d) and Pointe-Noire (c).

In order to assess the degree of parasite genetic diversity between urban and peri-urban areas, and so determine whether there is a correlation with entomological inoculation rate, we compared the number and types of parasites carrying different haplotypes of the *msp1* gene (Figure 3 panels A and B). We found that there was no significant difference in haplotype diversity (*h*) between the two regions (peri-urban region, *h* = 0.87, standard error (SE)  = 0.01; urban area, *h* = 0.88, SE = 0.02, Student’s two-tailed t-test, *P* = 0.81, d.f = 159). Furthermore, we found that there was no significant difference in the genetic *structure* of the two populations; the percent similarity (*Ps*) of *msp1* haplotypes between the urban and peri-urban regions was 0.84, which did not differ from expectation given random mixing between the two sites (95% range of permutation, 0.73–0.88), suggesting that the same parasite population is shared between the two areas. In order to assess whether *msp1* is a valid marker for population genetic structure that may indicate whether populations are discrete or mixed, we assessed the *msp1* genetic structure for two further sites within the Republic Of Congo, for which it was assumed that there is no parasite population mixing due to geographical isolation. These sites were Pointe-Noire (380 kilometers West of Brazzaville), and Gamboma (250 km to the North of Brazzaville). As there were no parasite population genetic structure differences between the urban and peri-urban regions, the percentage similarity was determined for both Point-Noire and Gamboma against a combined “Brazzaville” population. The haplotype pattern was less similar between Brazzaville and Pointe-Noire (Ps = 0.71, 95% range of permutation, 0.73–0.88) and Brazzaville and Gamboma (Ps = 0.77, 95% range of permutation, 0.70–0.86), than it was between the urban and peri-urban regions of Brazzaville.

### Population structure, parasite population genetic diversity and linkage disequilibrium analysis based on microsatellite markers

In order to determine whether the parasites in circulation in the peri-urban and urban areas were discrete populations or in panmixis, an *F_ST_* value was determined based on the microsatellite markers analyzed using FSTAT v.2.9.4. *F_ST_* for the combined population was 0.004, (SE 0.005), a very low value indicative of panmixia. There was no significant population differentiation between areas (G-test, 10,000 randomizations, *P* = 0.1). *h* was calculated for the two populations based on the mean genetic diversity of all microsatellite loci using LIAN v3.5. For the urban area, *h = *0.81, SE = 0.04, and for the peri-urban area, *h* = 0.83, SE = 0.04. The effective population sizes (*N_e_*) based on these *h* values were 6703 (95% CI, 2880–15,269) for the urban area, and 7676 (95% CI, 3299–17,486) for the peri-urban area. Individual values of *h* were also determined for each microsatellite loci as above, and are shown in Table 1. There were no significant differences in the *h* values of any loci between the two areas.

The degree of linkage disequilibrium was determined for both populations using LIAN v3.5. For the urban area, *I^S^_A_* = −0.0034 (*P = *0.585), and for the peri-urban area *I^S^_A_* = −0.0119 (*P = *0.786) indicating the complete absence of linkage disequilibrium in both areas. Pair-wise tests of linkage disequilibrium between pairs of microsatellite markers were performed using FSTAT v.2.9.4. There was no significant linkage between the majority of markers for either the urban or peri-urban areas, with the exceptions of TA53 (Chromosome (Chr) 9) and Poly-α (Chr 4) in the peri-urban area and in the combined population (considering both the peri-urban and the urban areas as one population) (*P*<0.01); TA60 (Chr 13) and Poly-α, also in the peri-urban area (*P* = 0.05); TA60 and TA53 in the urban area (*P* = 0.01); and TA43 (Chr14) and TA81 (Chr5) in the combined population (*P* = 0.03). There were no significant differences in percentage similarities (*Ps*) between samples from the urban and the peri-urban regions for each individual marker (Table 1).

### Allele frequencies of genes associated with resistance to chloroquine and pyrimethamine

The prevalence of drug-resistance associated mutations in the *crt* and *dhfr* genes, linked to resistance to pyrimethamine and chloroquine respectively, were assessed by PCR amplification and direct sequencing of products. For *dhfr*, we found a high proportion of parasites from both regions carried mutations at amino acid positions 51, 59 and 108, but that the 164 (I → L), mutation, known currently to be rare in Africa, was not observed. For *crt*, a high prevalence of parasites carrying the amino acid position 76 (K → T) was observed in both areas. Allele frequencies were determined using a maximum likelihood algorithm carried out using *MalHaploFreq* [45], and are shown in Table 4. We found that there was a significant difference in the allele frequencies at the *crt* locus between the urban and peri-urban areas, with significantly fewer wild-type parasites in circulation in the peri-urban area (log likelihood ratio test χ^2^ = 5.19, *P* = 0.023, d.f. = 1). There was also a significant difference in the allele frequencies of *dhfr* mutations between the two areas, with the highly resistant triple mutant more common in the peri-urban area (Log Likelihood ratio test χ^2^  = 19.4, *P*<<0.01, d.f. = 4

**Table 4:** Allele frequencies at the *dhfr* and *crt* loci for the urban and peri-urban areas, estimated using MalHaploFreq based on prevalence data combined with the number of clones per sample (determined by haplotyping *msp1*).

Allele	Frequency (95% CI)		*P*-value
*dhfr*	Peri-urban	Urban	
NCSI (wild type)	0.02 (0.00–0.05)	0.02 (0.00–0.07)	0.001
ICNI (double mutant)	0.29 (0.21–0.39)	0.36 (0.24–0.49)	
NRNI (double mutant)	0	0.13 (0.06–0.23)	
IRNI (triple mutant)	0.68 (0.59–0.77)	0.50 (0.37–0.63)	
*crt*			
CVMNK (wild type)	0.03 (0.01–0.07)	0.12 (0.06–0.20)	0.023
CVIET (mutant)	0.97 (0.92–0.99)	0.88 (0.79–0.94)	

*P*-values were determined using log-liklelihood tests.

## Discussion

Our results indicate that the *Plasmodium falciparum* parasite population in contiguous areas of urban and peri-urban Brazzaville, Republic of Congo circulates freely between both areas, and forms one population. Within this setting, we found no differences in the multiplicities of infections (MOI) of patients, parasite genetic diversity or linkage disequilibrium between the two areas, despite the large variation in the intensity of transmission between them (2–12 infective mosquito bites per person per year (ib/p/y) for the urban area, and ≈ 50 ib/p/y in the peri-urban area). It seems very likely that the relatively low transmission rates in the urban area result in a lower degree of immunity in the population residing there in comparison to those residing in the peri-urban area. This is supported by our data in two ways; firstly, by the larger proportion of patients displaying sub-microscopic parasite infections in the peri-urban area (20.1% of all PCR positive patients, compared with 11% in the urban area), and secondly by the fact that mean age of the sub-microscopic carriers was higher in the urban than in the peri-urban area (21.0 vs 15.4, respectively, although this was not significant, probably due to the low numbers of sub-microscopic carriers in the urban area). Increased vectorial capacity is thought to lead to higher strain diversity [10], [55], with the result that it is expected that greater parasite genetic diversity will be found in areas with higher transmission rates. However, our results show that parasite genetic diversity is uniform throughout an area with varying transmission rates, presumably due to gene flow within a population in panmixis. As immunity to malaria is thought to be acquired in a strain-specific manner [6], [7], [8], then, given homogeneity of parasite strain diversity within a given area, transmission intensity becomes the major factor for determining the rate at which immunity is achieved against the parasite population circulating within it. One possible consequence of increasing urbanisation is that decreasing transmission intensity may lead to a decrease in parasite genetic diversity, and so increase the rate at which immunity may be achieved by the population. However, our results indicate that parasite genetic diversity may not alter significantly, when the parasite population extends to the surrounding peri-urban areas, which seems the likely situation when considering the process of urbanisation, in which highly urbanized centers extend out into the less urbanized peripheries. This suggests that the acquisition of immunity in urban areas will take longer than it will in peri-urban and rural areas, and will lead to increased risk of symptomatic malaria for a longer period of childhood for those living there. Our results are in agreement with other surveys of the relationship between transmission rates and parasite genetic diversity [15], [16], [17], but in disagreement with others [12], [13]. In most of these studies, regions separated by large distances were compared; whereas our study is the first to compare contiguous regions where the potential of genetic transfer between high and low transmission areas exists.

We found significantly greater selection of parasites carrying drug-resistance associated alleles of *dhfr* and *crt*, linked to SP and CQ resistance, respectively, in the peri-urban area compared to the urban area. This result fits with the predictions of some theoretical models that drug resistance will be selected predominantly in high transmission areas [19], and with empirical evidence of the same trend from studies conducted in Western Uganda [34]. In these latter studies, the prevalence of *dhfr* mutants increased directly with transmission intensity, while the prevalence of *crt* mutations, whilst highest at high transmission areas, and lower at intermediate levels, increased again at the low transmission extreme. It seems likely that the “low” transmission setting (the urban area) in this study is analogous to the intermediate setting of the Ugandan study in terms of transmission intensity, and so the two data sets concur.

Our data does not appear to support the hypothesis that increased host immunity associated with high transmission areas can act as a “refuge” for wild type parasites [20], at least not in regions with a parasite pool shared between areas of low and high transmission. This hypothesis is based on a number of very reasonable assumptions; i) that high host immunity may reduce the selection advantage of resistant parasites in the presence of drugs [27], ii) that increased parasite genetic diversity leads to more clones per infection, and a greater opportunity for intra-host competition to select for sensitive parasites in the absence of drugs due to fitness costs associated with resistance [24] iii) that at high levels of population immunity, less antimalarial drugs are used per infection due to host immunity limiting the severity of the symptoms of malaria [20], [56]and iv) that in high transmission areas, higher recombination rates lead to faster breakdown of drug resistance caused by multi-loci mutations. In our study, MOI and parasite genetic diversity was not significantly different between the high and low transmission regions, presumably due to the sharing of the panmitic parasite pool between them. Thus, it is not likely that there is differential selection pressure mediated by intra-host competition between these areas. Similarly, we found no differences in linkage disequilibrium between areas, indicative of comparable rates of recombination, again probably due to the shared parasite population, thus there should be equal opportunities for the break-up of multi-locus resistance genotypes in both areas. We did not directly measure drug usage in both areas, so we do not know if there is less drug use in the peri-urban area compared to the urban area (as hypothesized in point iii), above), although this seems unlikely given that almost equal proportions of patients from both areas presenting to the health centers with fevers had taken some form of anti-malarial treatment themselves at home prior to presentation (Table 3). Given this, we consider that the most parsimonious explanation for the differences in the frequencies of drug resistance associated alleles of *dhfr* and *crt* between the urban and peri-urban areas is due primarily to greater drug pressure in the peri-urban area due to greater parasite prevalences. Thus our data suggests that urbanisation may lead to a decrease in the selection pressure for drug resistance by driving down transmission rates while not affecting parasite genetic diversity.

An alternative explanation for the observation of greater selection for drug resistance in the high transmission areas is that although moderately high levels of transmission may indeed provide a refuge for wild type parasites [20], [56], at very high levels of transmission, a threshold may be crossed at which point the resistant parasites again gain a selection advantage. This might happen when the duration of infections increases to the point that any fitness incurred by the drug resistant parasite is counter-balanced by the disadvantage of being sensitive to the drugs still applied to the population [23]. It may well be that the field setting considered here is more of a moderate versus very high transmission, than of a low versus high transmission.

In conclusion, we have shown, using a field setting in which contiguous urban and peri-urban areas sharing a parasite population but with very different levels of malaria transmission intensity, that urbanisation is likely to lower transmission rates without affecting parasite genetic diversity, and that this may lead to a reduced pressure for the selection of drug resistance. Targeting peri-urban areas with appropriate treatment strategies would, therefore, be important in minimizing the risk of the selection of drug resistance.
